# Whole genome resequencing data for rock pigeon (*Columba livia*)

**DOI:** 10.1186/s13104-021-05718-1

**Published:** 2021-08-09

**Authors:** Ali Esmailizadeh, Hamed Kharrati-Koopaee, Hojjat Assadoullahpour Nanaei

**Affiliations:** 1 Department of Animal Science, Faculty of Agriculture, Shahid Bahonar University of Kerman, PB 76169-133, Kerman, Iran; 2 Institute of Biotechnology, Shiraz University, Shiraz, Iran; 3 Key Laboratory of Animal Genetics, Breeding and Reproduction of Shaanxi Province, College of Animal Science and Technology, Northwest A&F University, Yangling, 712100 China; 4 State Key Laboratory of Genetic Resources and Evolution, Kunming Institute of Zoology, Chinese Academy of Sciences, No. 32 Jiaochang Donglu, Kunming, 650223 Yunnan China

**Keywords:** Rock pigeon, Navigation, Genome analysis

## Abstract

**Objective:**

Navigation is the most important feature of homing pigeons, however no integrated response to genetic mechanism of navigation has been reported. The generated data herein represent whole-genome resequencing data for homing pigeon and three other breeds of rock pigeons. Selective sweep analysis between homing pigeon and other breeds of rock pigeon can provide new insight about identification of candidate genes and biological pathways for homing pigeon ability.

**Data description:**

Whole-genomes sequence data related to 95 birds from four breeds of rock pigeons including, 29 feral pigeons, 24 Shiraz tumblers, 24 Persian high flyers and 18 homing pigeons were provided. More than 6.94 billion short reads with coverage (average ≈7.50 x) and 407.1 Gb data were produced. Whole genome sequencing was carried out on the Illumina Hiseq 2000 platform using a 350 bp library size and 150 bp paired-end read lengths. The whole genome sequencing data have been submitted at the NCBI SRA Database (PRJNA532675). The presented data set can provide useful genomic information to explain the genetic mechanism of navigation ability of homing pigeons and also testing other genetic hypothesis by genomic analysis.

## Objective

Domestic pigeon (*Columba livia domestica*) is a frequent bird around the world that was derived from the rock pigeon. They are native to Europe, North Africa, the Middle East, and South Asia. [1]. *C. livia* is known as an ideal model for different investigation such as ecology, genetics, physiology, behavior, and anatomical diversity [2]. The geographic origins of pigeon breeds have not been well described. However, it has been demonstrated that the origins of major breed groups of pigeons go back to the Middle East and North America [3]. Like other domestic animals, natural and artificial selections have remarkably impacted the genetic evolution in pigeons [4]. More than 350 breeds of pigeons have been reported and about 300 species of pigeons from family Columbidae are kept as pet [1]. For several reasons such as food source, decoration and fly sporting, pigeons have been kept for a long time. However, navigation is the most important feature of pigeons. Homing pigeon (also called racing homer) is able to navigate for finding the home’s way. This ability of pigeons played a vital role in wars to transfer the news of wars. Several investigations have been attempted to explain the mechanism of navigation in pigeons, but it has been remained as a puzzle [1, 3]. At present, several hypotheses have been proposed for the mechanisms of navigation ability in domestic pigeons, the most important of them are summarized in the following statements. The sun (solar compass) [5], the earth's magnetic (magnetic compass) [6], the olfactory and visual pathways [7, 8] and the specialized development of the hippocampus in brain [3]. Among these theories, the orientation of pigeons using the earth's magnetic field, called magnetoreception, is more considered as an environmental factor [9]. It also should be noted that hippocampus contribute to navigation in homing pigeons. Hippocampus is one of complex brain structure and has a critical role in learning and memory [10]. We carried out selective sweep analysis by generating 95 whole genomes sequence of different breeds of pigeons and several candidate genes and biological pathways were reported for homing pigeon ability [10]. The presented data set can provide interesting and applied resources to understand the genetic mechanism of navigation ability of homing pigeons and also examination of other associated hypothesis by genomic analysis.

## Data description

For selective sweep analysis, we collected 95 blood samples of rock pigeon breeds, including 29 feral pigeons, 24 Shiraz tumblers, 24 Persian high flyers and 18 homing pigeons. In this way, more than 6.94 billion short reads with coverage (average **≈**7.50 x) and 407.1 Gb data were generated [10] (Table 1). The collected rock pigeon’s breeds show different ability in navigation. As an example homing pigeon and feral pigeon have the strongest and weakest navigation ability and also Persian high flyer and Shiraz tumblers have the same navigation ability. The samples were obtained from four regions (Shiraz, Tehran, Kerman, and Marvdasht) in Iran. DNA was provided by phenol–chlorroform protocol. Agarose gel (1%) and absorption ratio 260/280 (nm) were applied to evaluation of the extracted DNA. Library size 350 bp and paired-end short read length 150 bp were generated by Illumina Hiseq 2000 [10]. Btrim (version: 2.0) was used to adaptors trimming and quality control of short reads. Clean short reads were aligned by default parameters of BWA-MEM (http://bio-bwa.sourceforge.net) against the pigeon reference genome (assembly accession: GCF_000337935.1) [10, 11]. SortSam and MarkDuplicates in Picard-tools-1.56 (http://broadinstitute.github.io/picard), was utilized to sort bam files and mark duplicates. SAMtools program (v0.1.19-44428cd) was applied to index the mapping files. Local realignment and base quality recalibration were performed by GATK (v2.6-4-g3e5ff60). The UnifiedGenotyper scripts of GATK was utilized for SNPs detection, and also SNPs filtration was carried out by the VariantFiltration in GATK. Annotation of SNPs were reported by SnpEff software (v4.3T) [10]. Around 20.6 million single nucleotide polymorphisms (SNPs) were reported after quality filtering of the detected SNPs, with ~7.6 × average sequence depth for each individual. In order to identify the associated selective sweep with homing ability between racing homer pigeon and residual breeds of pigeon, the genome-wide distributions of fixation index (Fst) was estimated based on the previous explained method [12]. The outcomes of signature selection analysis showed GSR gene (encoding glutathione-disulfide reductase) might be considered as result of positive selection in the homing pigeon. Gene expression analysis uncovered that GSR was highly expressed in the wattle and visual pigment cell layer, and displays increased expression levels in the homing pigeon. Our finding provides new insight about importance of the hippocampus for homing ability, and the potential role of GSR in pigeon magnetoreception.

**Table 1:** Overview of whole-genome sequence data files of four breeds of rock pigeons

Label	Name of data file/data set	File type (file extension)	Data repository and identifier (DOI or accession number)
Bioproject	*Columba livia* Genome sequencing	Fastq (fq.gz)	https://identifiers.org/ncbi/bioproject:PRJNA532675 [13]
Data set 1	s76_KIZ-FER1/ Feral rock pigeon	Fastq (fq.gz)	NCBI SRA Database https://identifiers.org/ncbi/insdc.sra:SRR10102989 [14]
Data set 1	s107_KIZ-FER29/ Feral rock pigeon		NCBI SRA Database https://identifiers.org/ncbi/insdc.sra:SRR10102958 [15]
Data set 1	s86_KIZ-FER10/ Feral rock pigeon		NCBI SRA Database https://identifiers.org/ncbi/insdc.sra:SRR10102979 [16]
Data set 1	s92_KIZ-FER16/ Feral rock pigeon		NCBI SRA Database https://identifiers.org/ncbi/insdc.sra:SRR10102972 [17]
Data set 2	s26_KIZ-HOP1/ Homing Pigeon	Fastq (fq.gz)	NCBI SRA Database https://identifiers.org/ncbi/insdc.sra:SRR10103035 [18]
Data set 2	s37_KIZ-HOP10/ Homing Pigeon		NCBI SRA Database https://identifiers.org/ncbi/insdc.sra:SRR10103025 [19]
Data set 2	s47_KIZ-HOP17/ Homing Pigeon		NCBI SRA Database https://identifiers.org/ncbi/insdc.sra:SRR10103017 [20]
Data set 2	s48_KIZ-HOP18/ Homing Pigeon		NCBI SRA Database https://identifiers.org/ncbi/insdc.sra:SRR10103016 [21]
Data set 3	s1_KIZ-PHF1/ Persian high flyer	Fastq (fq.gz)	NCBI SRA Database https://identifiers.org/ncbi/insdc.sra:SRR10103052 [22]
Data set 3	s8_KIZ-PHF8/ Persian high flyer		NCBI SRA Database https://identifiers.org/ncbi/insdc.sra:SRR10102985 [23]
Data set 3	s23_KIZ-PHF22/ Persian high flyer		NCBI SRA Database https://identifiers.org/ncbi/insdc.sra:SRR10103038 [24]
Data set 3	s25_KIZ-PHF24/ Persian high flyer		NCBI SRA Database https://identifiers.org/ncbi/insdc.sra:SRR10103036 [25]
Data set 4	s51_KIZ-SHT1/ Shiraz tumbler	Fastq (fq.gz)	NCBI SRA Database https://identifiers.org/ncbi/insdc.sra:SRR10103015 [26]
Data set 4	s66_KIZ-SHT16/ Shiraz tumbler		NCBI SRA Database https://identifiers.org/ncbi/insdc.sra:SRR10102999 [27]
Data set 4	s68_KIZ-SHT18/ Shiraz tumbler		NCBI SRA Database https://identifiers.org/ncbi/insdc.sra:SRR10102997 [28]
Data set 4	s74_KIZ-SHT24/ Shiraz tumbler		NCBI SRA Database https://identifiers.org/ncbi/insdc.sra:SRR10102990 [29]

The whole genome sequencing data was submitted at NCBI SRA Database with accession number PRJNA532675. A total 95 whole genome sequence files were generated for four different breeds of rock pigeons including feral rock pigeons (29 samples), homing pigeon (18 samples), Persian high flyer (24 samples) and Shiraz tumbler (24 samples). This Table shows the link for the bioproject and also four links were showed for each breeds of rock pigeons

## Limitations

The number of the breeds of rock pigeon collected and their genomes sequenced is a limitation of our investigation. We could only obtain four breeds for whole genome sequencing. In addition, it should be noted that, we produced the short-reads with a mean coverage of about 7.50× and probably, the data set could not support some genomic analyses.

## Data Availability

The whole genome sequence data described herein have been deposited in NCBI database as the sequence read archive (SRA) format (https://www.ncbi.nlm.nih.gov/sra/?term=PRJNA532675) under the accession number of PRJNA532675. Please see Table 1 and the references [13–29] for details and links to the data.

## Abbreviations

Gb: Giga byte
NCBI: The National Center for Biotechnology Information
BWA: Burrows-Wheeler aligner
SNP: Single nucleotide polymorphism
GATK: Genomic analysis toolkit
bp: Base pair
DNA: Deoxyribonucleic acid
